# Supplementary material for: WordCommentsAnalyzer: A windows software tool for qualitative research
Source: F1000Res. 2018 Sep 4;7:536. Originally published 2018 May 3. [Version 2] doi: 10.12688/f1000research.14819.2 (PMC6137411; doi:10.12688/f1000research.14819.2)
Supplement: Supplementary file 3 [file f1000research-7-17614-s0002.tgz › b2a79496-4ada-47c4-9682-a99ce867836d.zip!/Eight Iranian Health Journals/The Journal of Toloo-e-Behdasht_18.docx]

© The Journal of Toloo-e-Behdasht. This work is licensed under CC BY 4.0 (https://creativecommons.org/licenses/by/4.0/).

Volume & Issue: دوره 14 - شماره 3

Title: بررسی اثر بخشی گندزداهای رایج بر میکروارگانیسم‌های جدا شده از قسمت‌های مختلف بخش سوختگی بیمارستان سوانح و سوختگی شهید صدوقی یزد در سال 1390

http://tbj.ssu.ac.ir/article-1-1673-fa.html

چکیده مقدمه: گندزدایی فرآیندی است که طی آن میکروارگانیسم‌های بیماریزای سطوح غیر‌زنده از بین می‌روند، انتخاب ضدعفونی‌کننده مناسب و بکارگیری روش‌های استاندارد گندزدایی می‌تواند در کاهش عفونت-های بیمارستانی نقش مؤثری داشته باشد. هدف این مطالعه ارزیابی قدرت اثر‌بخشی گندزدا‌های رایج بر روی میکروارگانیسم‌های جدا‌شده از قسمت‌های مختلف بخش سوختگی بیمارستان سوانح سوختگی شهید ‌صدوقی یزد می‌باشد. روش بررسی: در این تحقیق توصیفی-تحلیلی از قسمت‌های مختلف بیمارستان سوانح سوختگی بطور تصادفی جمعاً 240 نمونه میکروبی گرفته شد (برای هر گندزدا 30 نمونه قبل و30 نمونه بعد از گندزدایی از مکان‌های مشترک). در آزمایشگاه میکروب شناسی دانشگاه علوم پزشکی شهید صدوقی یزد، نمونه‌ها بر روی محیط آگار‌خون دار و EMB آگار کشت داده شده، توسط تست‌های بیوشیمیایی تعیین هویت گردیده و تعداد کلنی‌ها مشخص شد. گندزدا‌های دکونکس AF 50، دسکوسید، اپی‌مکس SC و سیلوسپت مورد بررسی قرار گرفت. داده‌ها با استفاده از آزمون آماری T-Test زوجی آنالیز گردید. یافته‌ها: میانگین باکتری‌های جدا‌شده از 4 قسمت بخش سوختگی بیمارستان، قبل از گندزدایی با دکونکس AF 50، دسکوسید، اپی مکس SC و سیلوسپت، به ترتیب 49/33737، 18/25784، 65/35085 و77/30504 و بعداز گندزدایی به ترتیب 3/14، 42/18569، 15/2 و 63/5 بود که در همه گندزداها کاهش آلودگی تفاوت معنی‌داری داشت(05/0p<). شایع‌ترین میکروارگانیسم‌های‌های جدا‌شده سودوموناس آئروژینوزا، کلبسیلا پنومونیه، استافیلوکوکوس اپیدرمیدیس،گونه‌های انتروباکتر، اشریشیاکلی و مخمر بود. نتیجه‌گیری: نتایج نشان داد همه گندزداهای مورد مطالعه، بر میکروارگانیسم‌های جداشده مؤثر بوده و همچنین بین میانگین تعداد باکتری‌ها قبل و بعد گندزدایی اختلاف معنی‌داری مشاهده و مؤثرترین گندزدا اپی‌مکس SC شناخته شد.

Title: میزان آشنایی اعضای نهادهای محلی استان‌های شمالی ایران از ضوابط طرح پزشک خانواده و بیمه روستایی در سال 1391

http://tbj.ssu.ac.ir/article-1-1674-fa.html

مقدمه: اعضای نهادهای محلی با نفوذ و منابعی که در اختیار دارند می‌توانند در اعتلای برنامه‌های سلامت نقش موثری را ایفا نمایند. همکاری مطلوب نهادهای محلی مستلزم آگاهی و شناخت آنها از اهداف و ضوابط تعیین شده می‌باشد. این مطالعه به منظور بررسی سطح آشنایی اعضای نهادهای محلی از ضوابط طرح پزشک خانواده و بیمه روستایی در استان‌های شمالی ایران انجام گرفته است. روش بررسی: مطالعه از نوع توصیفی به روش مقطعی بوده که در شش ماهه دوم سال 1391 انجام گرفت. 25 درصد مراکز مجری طرح پزشک خانواده و بیمه روستایی در سه استان گلستان، مازندران و گیلان، مجموعا 139 مرکز از 552 مرکز مجری بصورت تصادفی منظم به عنوان خوشه‌های پژوهش انتخاب و در هر مرکز منتخب اعضای شورای حل اختلاف، شورای اسلامی، شورای بهداشتی، دهیار، مدیر و معلم بومی در دسترس مورد بررسی قرار گرفتند. ابزار جمع‌آوری داده‌ها پرسشنامه محقق ساخته بوده که روایی و پایایی (84%) آن مورد تائید قرار گرفت. داده‌ها در18 SPSS با آزمون‌های مرتبط آماری در سطح معنی-داری 05/0>P تحلیل شد. یافته‌ها: از بین 601 نفر عضو نهادهای محلی مطالعه شده، 521 نفر (7/86 درصد) مرد بودند. جوان‌ترین فرد 19 ساله و مسن‌ترین 81 ساله که میانگین و انحراف معیار سن کل افراد 7/9±45 سال بود. از نظر تحصیلات، بیشتر افراد (1/47 درصد) مدرک دانشگاهی داشتند.. از کل 27 نمره، میانگین آشنایی دهیار 4±4/12، اعضای شورای بهداشتی 7/3±6/11، اعضای شورای اسلامی 1/4±4/11، مدیر بومی 3/3±6/10، اعضای شورای حل اختلاف 1/5±5/10 و معلم بومی 9/4±8/8 بوده است. بین سطح آشنایی و عضویت در هیات امناء مرکز، جنس، نوع نهاد محلی رابطه معنی‌دار وجود داشت (05/0>P). میزان آشنایی با تعداد مراجعه به خانه بهداشت و مرکز بهداشتی درمانی رابطه مستقیم معنی‌دار وجود داشت (05/0>P). بین میزان آشنایی و تحصیلات، استان‌های محل سکونت و تاهل رابطه معنی‌دار وجود نداشت (05/0

Title: افزایش اختلالات تغذیه و نقش آنها در بروز اضطراب اجتماعی اندام درمردان فعال مسن

http://tbj.ssu.ac.ir/article-1-1675-fa.html

مقدمه: اختلالات تغذیه نقش مهمی را در سلامت جسمی و روانی سالمندان دارای فعالیت ورزشی ایفا می کند. انرژی مورد نیاز بدن در مردان مسن به دلیل تغییر در ترکیبات بدن و کاهش متابولیسم پایه از یک طرف و کاهش فعالیت بدنی از طرف دیگر کاهش می یابد. هدف از انجام این تحقیق بررسی ارتباط بین اختلالات خوردن و اضطراب اجتماعی اندام در مردان مسن فعال دارای فعالیت ورزشی بود. روش بررسی: جامعه آماری این پژوهش کل مردان مسن فعال دارای فعالیت ورزشی شهرستان بابل با حداقل دو سال سابقه ورزشی بودند که در پارک های این شهرستان به ورزش می پرداختند. 100نفر بصورت تصادفی بعنوان نمونه آماری انتخاب شدند. این افراد دو پرسشنامه اختلالات خوردن گارنر و گارفینگل(پایایی85/0،روایی86/0) و مقیاس اضظراب اجتماعی فیزیکی مکنزی(پایایی85/0 و روایی81/0) را تکمیل کردند. برای تجزیه تحلیل داده های توصیفی از جمله آزمون های آماری میانگین، انحراف معیار و تحلیل رگرسیون استفاده شد. یافته ها: نتایج نشان داد که بین اختلالات خوردن و اضطراب اجتماعی اندام رابطه مثبت و معناداری وجود دارد (001/0P=). علایم بی اشتهایی عصبی و پراشتهایی عصبی قوی ترین متغیرها در پیش بینی علایم اضطراب اجتماعی اندام می باشند(001/0P>). مولفه بی اشتهایی عصبی نسبت به پراشتهایی عصبی سهم بیشتری از اختلالات خوردن را در مردان مسن فعال داشت. نتیجه گیری: این تحقیق پیشنهاد می کند که با تنظیم منظم عادات غذایی مردان فعال مسن و فعالیت بدنی آنها می توان نگرش آنها را در حضور در اجتماع بصورت مثبت تغییر داد. تا آنها با تحرک کافی و ورزش منظم دید مثبتی از اندام خود داشته باشند.

Title: بررسی کارایی سیستم لجن فعال هوادهی گسترده با سیکل متناوب (ICEAS) در حذف همزمان نیتروژن و فسفر از فاضلاب بیمارستانی

http://tbj.ssu.ac.ir/article-1-1676-fa.html

مقدمه: فاضلاب بیمارستانی حاوی انواع مواد مغذی و بطور مشخص نیتروژن و فسفر می‌باشد که در صورت تخلیه به محیط، منجر به ایجاد مشکلاتی دراکوسیستم‌های آبی و جانوری و در نهایت سلامت انسان می گردد. هدف از انجام این مطالعه تعیین کارایی سیستم لجن فعال هوادهی گسترده با سیکل متناوب (ICEAS) در حذف همزمان نیتروژن و فسفر از فاضلاب بیمارستانی است. روش بررسی: در این مطالعه توصیفی – مقطعی ابتدا یک راکتور لجن فعال هوادهی گسترده با سیکل متناوب (ICEAS) طراحی گردید. با گذشت دوره سازگاری میکروارگانیسم‌ها و شروع مراحل اصلی بارگذاری سیستم، اقدام به نمونه ‌برداری از فاضلاب در حین اختلاط بدون هوادهی، ورودی و خروجی سیستم گردید. از 180 نمونه جمع آوری شده، پارامترهای TN, TKN, NH4-N, organic-N, NO2-, NO3-, TP مورد سنجش قرار گرفتند. تجزیه تحلیل نتایج با کمک نرم افزارDesign expert انجام شد. یافته‌ها: راندمان حذف فسفر کل ، ازت آلی، ازت آمونیاکی، کل ازت کجلدال و کل ازت به ترتیب 90%-26%، 3/95% - 16%، 6/94% - 27%، 95% - 30% و 90%-5/17% حاصل شد. غلظت نیتریت و نیترات در خروجی سیستم بترتیب به mg/l 16/3-36/0 و mg/l 3/6-24/1 رسید. حداکثر راندمان حذف پارامترهای مذکور در بالاترین مقادیر زمان‌ماند هیدرولیکی (6 ساعت) و غلظت لجن تانک هوادهی (mg/l 6000) بدست آمد. نتیجه‌گیری: سیستم ICEAS با وجود این‌که در زمره فرایندهای اختصاصی حذف فسفر و نیتروژن قرار ندارد اما در شرایط مناسب راهبری قادر به حذف عناصر فوق با راندمان بالا می باشد. از این سیستم می‌توان جهت تصفیه فاضلاب بیمارستانی در مقیاس واقعی استفاده نمود.

Title: بررسی فراوانی سالمونلا و استافیلوکوکوس اورئوس در سالاد الویه صنعتی تولیدی در شهر یزد در سال1392

http://tbj.ssu.ac.ir/article-1-1677-fa.html

مقدمه: تأمین نیازهای غذایی جوامع بشری با توجه به رشد روز افزون جمعیت از مشکلاتی است که بویژه کشورهای در حال توسعه با آن مواجه هستند. با توجه به تغییر شرایط اجتماعی و اقتصادی جامعه و عادات غذایی جدید، فرهنگ استفاده از غذاهای آماده رایج گردیده است. سالاد اولویه یکی از غذاهای سرد متداول درکشور ما است که باتوجه به مغذی بودن در معرض خطر آلودگی می باشد به همین دلیل در این مطالعه کیفیت میکروبی سالاد الویه را از نظر حضور باکتری های سالمونلا و استافیلوکوکوس اورئوس مورد ارزیابی قرار دادیم . روش بررسی: در این مطالعه توصیفی تعداد 200 نمونه سالاد الویه از بازار مصرف تهیه شد و هنگام تهیه نمونه ها دمای محل نگهداری نیز اندازه گیری گردید. نمونه ها بلافاصله پس از تهیه، در شرایط استریل آزمایشگاه از نظر حضور سالمونلا طبق استاندارد ملی ایران به شماره 1810 و استافیلوکوکوس اورئوس طبق استاندارد ملی ایران به شماره1-6806 مورد آزمایش قرار گرفتند. و دالوها با استفاده از 11 Spss و آزمون Chi-Square تجزیه و تحلیل گردید. یافته ها: کلیه نمونه ها از نظر حضور سالمونلا منفی بودند. از 200 نمونه آزمایش شده 40 نمونه به باکتری استافیلوکوکوس اورئوس آلودگی داشتند و تعداد 160 نمونه فاقد آلودگی بودند. 30% نمونه های آلوده به استافیلوکوکوس اورئوس در دمای 4 تا 6 درجه سانتی گراد و 70% در دمای 6 تا 8 درجه سانتی گراد نگهداری می شدند. نتایج نشان داد که بین دمای محل نگهداری نمونه ها و موارد آلودگی مشاهده شده رابطه آماری معنی داری وجود دارد( 001/0 =P ). نتیجه گیری : با توجه به اهمیت سالمونلا و استافیلوکوکوس اورئوس در مسمومیت های غذایی و همچنین آلودگی قابل توجه نمونه ها به استافیلوکوکوس اورئوس به علت عدم رعایت موازین بهداشتی در حین تولید و یا آلودگی مواد اولیه و نیز افزایش آلودگی با افزایش دمای محل نگهداری ضرورت کنترل و نظارت دقیق مراکز عرضه و تولید مواد غذایی توسط واحدهای بهداشتی بیشتر مشخص می گردد.

Title: ارزیابی فرآیند جذب درحذف رنگ از پسآب‌های صنعتی

http://tbj.ssu.ac.ir/article-1-1678-fa.html

چکیده مقدمه: فرایندهای نوآورا نه برای تصفیه پسآب‌های صنعتی حاوی رنگ‌ها و فلزات سنگین اغلب برای کاهش سمیت این آلاینده‌ها به منظور رسیدن به استانداردهای تصفیه می‌باشد. اخیرا، تمرکز ویژه به فرآیندهای ابتکاری برای حذف فیزیکی و شیمیایی مثل فرایند جذب بر روی جاذب جدید، جذب زیست توده، فیلتراسیون غشایی، پرتوافکنی، و انعقاد الکتروشیمیایی صورت گرفته است. در این مطالعه ،توسعه‌های اخیر و کاربردی بودن روشهای مختلف تصفیه برای حذف رنگ از پساب صنعتی بررسی شد. روش بررسی: این مطالعه از 45 مقاله منتشر شده از سایت‌های مختلف بین سال‌های (2014-1997) استفاده شد که در اغلب آنها مزایا و محدودیت‌های کاربرد روش جذب سطحی ارزیابی شده است.در این مقالات، شرایط اصلی عملیاتی موثر بر فرایند جذب مانند pH، دما، غلظت آلاینده‌ها و مقدار جاذب در عملکرد تصفیه آلاینده رنگ ارزیابی شدند. نتیجه گیری: این مطالعه مروری نشان داد که فاکتورpH در اغلب مطالعات بطور متناوب مطالعه و تاثیر آن به طور گسترده برای تصفیه پساب‌های آلوده شده به رنگ بررسی شده بود. مطالعات نشان دادند که در آینده نزدیک، امیدوار کننده‌ترین روش برای تصفیه در سیستم‌های پیچیده فرایند جذب خواهد بود. فرایند جذب برای حذف آلاینده های آلی و بهبود فلزات در سیستم‌های یک راکتوری ناپیوسته کارایی خوبی نشان داده است. علاوه بر این، از فرآیندهای متعارف، جذب به عنوان یکی از مؤثرترین فنآوری برای تصفیه فاضلاب آلی و معدنی با غلظت آلاینده کمتر از 1000 میلی گرم / لیتر شناخته شده است. مهم است که توجه داشته باشیم که هزینه تصفیه کامل از آب آلوده شده متفاوت است و بسته به فرآیند بکار رفته و شرایط محلی دارد. به طور کلی، از عوامل کلیدی در انتخاب یک روش کاربردی بودن، سادگی طرح و مقرون به صرفه بودن آن است که فرایند جذب این مزایا را ارئه کرده و مناسب برای حذف رنگ از پساب‌ها است.

Title: بررسی وضعیت اجرای برنامه پزشک خانواده و انطباق آن با ابعاد سیاستگذاری شده از دیدگاه ارائه دهندگان خدمات در استان یزد

http://tbj.ssu.ac.ir/article-1-1680-fa.html

مقدمه: برنامه پزشک خانواده که یک برنامه اصلاحی برای دسترسی اقشار آسیب پذیر می باشد، فرآیندی سیاسی است وآگاهی و شناخت اهداف این فرآیند سیاستگذاری برای ارائه دهندگان خدمات از ضروریات می باشد. هدف از پژوهش حاضر، بررسی وضعیت اجرای پزشک خانواده با ابعاد سیاستگذاری شده از دیدگاه ارائه دهندگان خدمات در استان یزد بود. روش بررسی: روش مطالعه توصیفی تحلیلی و مقطعی بود. جامعه آماری شامل کلیه پزشکان خانواده و ماماهای شاغل در مراکز بهداشت استان یزد به تعداد 105 نفر بودند که به روش سرشماری کلیه آنها انتخاب شدند.برای جمع آوری دادهها از پرسشنامه محقق ساخته که روایی و پایایی آن مورد تأیید قرارگرفته بود، استفاده گردید. برای تجزیه و تحلیل دادهها در سطح آمار توصیفی از میانگین، انحراف معیار ، فراوانی و درصد و در سطح استنباطی ازآزمون‌های‌تی تک گروهی، نسبت، تی گروه‌های مستقل و آزمون استقلال استفاده شد. یافته‌ها: یافته‌ها نشان داد که ارائه دهندگان خدمات پزشک خانواده از بین ابعاد سیاستگذاری شده، وضعیت ابعاد اثربخشی، پایش، تناسب و تاثیرگذاری را بالاتر از حد متوسط و وضعیت ابعاد کفایت و کارایی را در حد متوسط ارزیابی نموده‌اند. نتیجه‌گیری: نتایج حاصل از یافته‌ها حاکی از آن بود که وضعیت ابعاد اثربخشی، پایش، تناسب و تاثیرگذاری مناسب و وضعیت ابعاد کفایت و کارایی در حد متوسط است، در بعد اثربخشی بین نظرات پزشکان و ماماها تفاوت معنی‌دار وجود داشت و درمورد سایر ابعاد تفاوت معناداری مشاهده نشد.

Title: بررسی و مقایسه سازگاری زناشویی، شادکامی و میل به طلاق در ازدواجهای سنتی و غیر سنتی شهر یزد

http://tbj.ssu.ac.ir/article-1-1681-fa.html

چکیده مقدمه:سازگاری زناشویی،شادکامی و متغیر پیش بینی کننده میل به طلاق از جمله ویژگیهای هستند که برای سنجش میزان موفقیت یک ازدواج کاربرد دارند از طرفی وجود دو سبک ازدواج سنتی و غیر سنتی و چالش موجود بین آنها در جامعه اهمیت مقایسه بین این دو سبک ازدواج را نشان می دهد لذاهدف این پژوهش مقایسه سازگاری زناشویی، شادکامی و میل به طلاق در ازدواج های سنتی و غیرسنتی شهر یزد بود. روش بررسی: جامعه آماری این پژوهش را کلیه زوجین شهر یزد تشکیل می داد. نمونه پژوهش شامل 240 نفر (130 مورد ازدواج سنتی و 110 مورد ازدواج غیر سنتی )بود که به روش نمونه گیری تصادفی خوشه ای انتخاب شدند.جهت گردآوری داده ها از پرسشنامه های سازگاری زناشویی لاک-والاس،شادکامی آکسفورد و میل به طلاق زارعی استفاده گردید.روش پ‍ژوهش حاضر از نوع غیر آزمایشی علی- مقایسه ای(پس رویدادی) بود و داده ها با استفاده از نرم افزار spss و آزمون آماری t مورد تجزیه و تحلیل قرار گرفت. یافته ها: نتایج نشان داد علی رغم تفاوت میانگین ها در شادکامی و سازگاری زناشویی بین زوجین با ازدواج سنتی و غیر سنتی (شادکامی بالاتر در ازدواج های غیرسنتی و سازگاری زناشویی بالاتر در ازدواج های سنتی)تفاوت معناداری بین آنها وجود ندارد(05/0 p>). ولی در مورد متغیر میل به طلاق نتایج نشان داد بطور معنی داری میل به طلاق در ازدواج های غیرسنتی بیشتر از سنتی می باشد(05/0 p<).همچنین از جهت عامل جنسیت؛ بین شادکامی، سازگاری زناشویی و میل به طلاق در بین زنان و مردانی که ازدواج سنتی داشته اند و شادکامی و میل به طلاق در بین زنان و مردانی که ازدواج غیر سنتی داشته اند تفاوت معنی داری وجود ندارد (05/0 p>).ولی سازگاری زناشویی در زنان و مردانی که ازدواج غیر سنتی داشته اند تفاوت معنی داری را نشان می دهد(05/0 p>)بطوریکه میزان سازگاری در مردان با ازدواج غیر سنتی بیشتر است. نتیجه گیری :سبک ازدواج(سنتی یا غیر سنتی) می تواند در تعیین شادکامی، سازگاری زناشویی و میل به طلاق بر روی زوجین تاثیر بگذارد.

Title: بررسی آگاهی و عملکرد مردان دارای همسران مبتلا به سندرم قبل از قاعدگی در خصوص علایم این سندرم در شهر یزد در سال 1392

http://tbj.ssu.ac.ir/article-1-1682-fa.html

مقدمه: سندرم قبل از قاعدگی PMS))Premenstrual Syndrome شامل نشانه های جسمی، روانی و عاطفی است که در طول اواخر چرخه قاعدگی آغاز شده و با شروع قاعدگی یا چند روز اول خونریزی رفع می شود، لذا این مطالعه با هدف تعیین آگاهی و عملکرد مردان دارای همسران مبتلا به سندرم قبل از قاعدگی، درخصوص علایم این سندرم در شهر یزد انجام گرفت. روش بررسی: این مطالعه توصیفی تحلیلی بر روی 240 زوج جوان مراجعه کننده به مراکز بهداشتی درمانی شهر یزد که به طور تصادفی و داوطلبانه انتخاب شدند، انجام شد. ابزار مورد استفاده در این پژوهش پرسشنامه غربالگری علایم قبل از قاعدگی P‏remenstrual Symptoms Screening Tool(PSST) و پرسشنامه محقق ساخته برای سنجش میزان آگاهی و عملکرد مردان درخصوص سندرم قبل از قاعدگی بود. روایی و پایایی پرسشنامه بعد از مطالعه مقدماتی تایید گردید. اطلاعات جمع آوری شده توسط نرم افزار آماری 18SPSS و با استفاده از آزمونهای آماری T-Test، ANOVA و ضریب هم بستگی مورد تجزیه و تحلیل قرار گرفت. یافته ها:میانگین نمره آگاهی مردان از علایم PMS که شامل میانگین نمرات آگاهی از علایم روانی، جسمی و راهکار های پیشگیری یا کاهش بروز علایم بود برابر4/7±51/26 از نمره 48و میانگین نمره عملکرد مردان در مورد PMS2/5±01/23 از نمره 32بود. بین نمره آگاهی و عملکرد مردان رابطه معنی دار وجود داشت(000/0P=). کمترین پاسخ صحیح به آگاهی از نشانه های روانی، جسمی و راهکارهای پیشگیری از PMS به ترتیب به تمایلات خودکشی، افزایش اشتها و کاهش وزن و کمترین عملکرد مربوط به گزینه (در دوران پیش از قاعدگی همسرم، هر چند ساعت ازسرکار به او تلفن زده و یادآور می شوم چیزی میان وعده بخورد) اختصاص داشت. نتیجه گیری: با توجه به نتایج توصیه می گردد برای دستیابی به آگاهی و تغییر رفتار نسبتا پایدار، بر کیفیت آموزش های لازم در خصوص نشانه های PMS مثل تمایلات خودکشی و خودآزاری، افزایش اشتها و ولع غذایی، راهکار کاهش وزن و توجه و یادآوری میان وعده به همسران تاکید گردد.

Title: طراحی مدل بر اساس تعهد سازمانی و وابستگی شغلی پزشکان در تخمین میزان خطاهای پزشکی در ویزیت های سرپائی

http://tbj.ssu.ac.ir/article-1-1684-fa.html

مقدمه: عوامل مختلفی از جمله وابستگی شغلی و تعهد سازمانی پزشکان باعث وقوع خطاهای پزشکی می شود. در این مطالعه مدلی برای تخمین وقوع خطا بر اساس ارتباط همزمان این دو متغیر بر وقوع خطا در ویزیت های سرپائی ارائه می شود. روش بررسی: در مطالعه میدانی114 پزشک، میانگین خطاها بدست آمدند. میزان وابستگی شغلی و تعهد سازمانی پزشکان بر اساس پرسشنامه عظیمی و پرسشنامه آلن و مایر ارزیابی شد. میانگین خطاهای رخ داده پزشکان بر اساس وضعیت همزمان تعهد سازمانی و وابستگی شغلی آنها در چهار گروه (گروه اول تعهد سازمانی بالا و وابستگی شغلی بالا، گروه دوم تعهد سازمانی پائین و وابستگی شغلی بالا، گروه سوم تعهد سازمانی بالا و وابستگی شغلی پائین وگروه چهارم تعهد سازمانی پائین و وابستگی شغلی پائین) توسط تستهای آنوا و شفه تحلیل شد. بر این اساس مدل نهایی طراحی گردید و اعتبارسنجی به روش داخلی انجام شد. یافته ها: میانگین خطای پزشکان گروه اول (12/0±32/0)،گروه دوم (09/0±51/0، گروه سوم (07/0±41/0)،گروه چهارم (12/0±50/0)، میانگین تعهد سازمانی پزشکان(30/12±50/79) و میانگین وابستگی شغلی پزشکان( 66/3±72/12) بود. آنوا (آزمونF 20/22و سطح معنی داری کمتر از 01/0) و آزمون شفه (سطحی معنی داری کمتر از 05/0) با عدم وجود صفر در سطح پائین و بالای همه گروه ها به جز بین دو گروه دوم و چهارم پزشکان مشاهده شد. آنوا تفاوت معنی دار میانگین خطاهای پزشکی چهار گروه پزشکان و شفه معنی دار بودن دو به دو گروه ها بجز گروه دوم و چهارم را تایید کرد. مدل ارائه شده دارای 6/73 درصد اعتبار بود. نتیجه گیری: برنامه ریزی و ارائه راهبردهایی جهت افزایش تعهد سازمانی و وابستگی شغلی پزشکان ،خطاهای پزشکی را کاهش می دهد. در این راستا مطالعه عوامل موثر بر تعهد سازمانی و وابستگی شغلی پزشکان برای مداخله مذکور الزامی می باشد.

Title: بیمه های بهداشتی درمانی حمایتی در دنیا: مطالعه تطبیقی کشورهای منتخب

http://tbj.ssu.ac.ir/article-1-1683-fa.html

مقدمه: بیمه‌های بهداشتی درمانی حمایتی یکی از مهمترین روش‌ها برای ارائه خدمات سلامت به مردم می باشند. با توجه به تجارب کشورهای مختلف در این زمینه هدف از انجام این مطالعه بررسی تطبیقی بیمه های بهداشتی درمانی حمایتی در کشورهای منتخب می باشد. روش بررسی: این مطالعه از نوع مطالعات تطبیقی می باشد. حوزه پژوهش شامل مدل های بیمه بهداشتی درمانی حمایتی در کشورهای مختلف و ایران می باشد. کشورهای مورد بررسی در این پژوهش را آمریکا، پاراگوئه، انگلستان، سوئد، فرانسه، تایلند، استرالیا و ژاپن تشکیل می دهند. که بر مبنای تقسیم بندی گاردن در تقسیم بندی نظام های بیمه ای انتخاب شده اند. تحلیل اطلاعات در محورهای سیاستگذاری، برنامه ریزی کلان، ساختار و معیار و مبنای پوشش انجام گرفته است. یافته ها: نتایج نشان داد که وزارت های بهداشت تولیت بیمه های درمانی را بر عهده دارند. در زمینه برنامه ریزی، برنامه ریزی در سطح ملی و منطقه ای پیشنهاد شده است و در بیشتر کشورها این برنامه ها به صورت غیرمتمرکز در شهرها و روستاهای هر منظقه اجرا می گردد. تمایل و گرایشی بیشتری برای سازماندهی دولتی در ارائه بیمه بهداشتی درمانی حمایتی وجود دارد. در بحث مبنای پوشش نیز عدالت و توجه به فقرا بیشترین تاثیر را دارد. نتیجه گیری: براساس نتایج مطالعه حاضر، طراحی مدل بیمه ای درمان حمایتی که تولیت آن در اختیار وزارت بهداشت و برنامه ریزی آن در سطح ملی و منطقه ای ولی اجرا در سطح مناطق باشد و از یک ساختار دولتی تبعیت کند و عامل فقر را شرط پوشش قرار دهد توصیه می شود.

Title: بررسی میزان تاثیر آموزش پیشگیری از سرطان پوست در معلمان شهر یزد

http://tbj.ssu.ac.ir/article-1-1685-fa.html

چکیده مقدمه: سرطان‌ها سومین عامل مرگ و میر در ایران محسوب می‌شوند و شایع‌ترین آن‌ها سرطان پوست است که در استان یزد از شیوع بالاتری نسبت به کشور برخوردار است. با توجه به نقش مهم معلمین و مدارس به عنوان جایگاه مناسب برای آموزش به دانش آموزان و خانواده آن‌ها این مطالعه با هدف تعیین میزان تاثیر آموزش پیشگیری از سرطان پوست در معلمان شهر یزد در سال 1391 صورت گرفت. روش بررسی: این مطالعه نیمه تجربی به صورت قبل و بعد به روش نمونه گیری تصادفی در میان 80 نفر از معلمان شهر یزد انجام گرفت. ابزار مورد استفاده پرسشنامه‌ای دارای روایی و پایای معتبر در چهار بخش اطلاعات جمعیت شناختی، آگاهی، نگرش و عملکرد بود که در دو مرحله در قبل و بعد از آموزش به صورت خود گزارش دهی تکمیل گردید. یافته‌ها: محدوده سن شرکت کنندگان 23 الی 51 سال با میانگین 22/6±87/36 بود. 3/77 درصد زن، 7/22 مرد بودند. میانگین نمره آگاهی، نگرش و عملکرد در بعد از مداخله آموزشی افزایش یافت (001/0>P). بعد از مداخله آموزشی با افزایش میزان نگرش شرکت کنندگان میزان عملکرد نیز افزایش پیدا کرد (001/0p=، **407/0r=) و مردها نیز نگرش بالاتری نسبت به زنان پیدا نمودند (04/0p=). نتیجه گیری: مداخله آموزشی انجام شده در آگاهی، نگرش و عملکرد بهداشتی معلمان شهر یزد در خصوص سرطان پوست به شکل معناداری موثر بود.
